# A case of accidental into-the-lens dexamethasone implant: watching or removing?

**DOI:** 10.1186/s12886-024-03538-y

**Published:** 2024-07-11

**Authors:** Maria Ludovica Ruggeri, Alberto Quarta, Rossella D’Aloisio, Lisa Toto, Rodolfo Mastropasqua

**Affiliations:** 1 Ophthalmology Clinic, Department of Medicine and Science of Ageing, University “G. D’Annunzio” Chieti- Pescara, via dei Vestini 31, Chieti, 66100 Italy; 2 Department of Neurosciences, Imaging and Clinical Sciences, University “G. d’Annunzio” Chieti-Pescara, Chieti, Italy

**Keywords:** Cataract, Ozurdex, Vitrectomy, Diabetic macular edema

## Abstract

**Background:**

To report a case of cataract surgery in unintentional Ozurdex (Allergan, Inc., Irvine, California, USA) injection into the lens.

**Case presentation:**

A 82-years old man reporting decreased visual acuity in his right eye came to our Ophthalmology service. Due to the clinical history, and on the basis of ophthalmoscopic and imaging examinations diabetic macular edema was diagnosed. Thus, intravitreal dexamethasone implant was scheduled and therefore performed. The following day Ozurdex appeared to be located into the lens. After careful evaluation and strict follow up examinations, due to the risks associated with the presence of the implant into the lens, phacoemulsification with Ozurdex removal and intraocular lens (IOL) implantation was scheduled and performed.

**Conclusions:**

In this case report we reported the surgical management of accidental into-the lens dexamethasone implant carefully taking into account the dexamethasone pharmacokinetic.

**Supplementary Information:**

The online version contains supplementary material available at 10.1186/s12886-024-03538-y.

## Introduction

Intravitreal dexamethasone implant (Ozurdex, Allergan, Inc., Irvine, California, USA) is a sustained-release biodegradable implant which has demonstrated overall favorable safety and tolerability profile. Although rarely, an accidental injection of the device into the lens may occur, potentially leading to complications such as cataract formation, intraocular pressure (IOP) raise and corneal decompensation.

We report a case of inadvertent into-the-lens dexamethasone implant treated with phacoemulsification, Ozurdex removal and lens implantation in order to safely remove cataract and allow monitoring the underlining retinal disease.

## Case description

An 82 years-old caucasian man came to our Ophthalmology service in March 2023 complaining about unilateral blurred vision in his right eye.

Best corrected visual acuity (BCVA) was 0.7 logMar in his right eye and 0.4 logMAR in his left eye, fundus examination showed diabetic macular edema in his right eye, thus, intravitreal dexamethasone 700 mcg implant was scheduled and then administered.

**Fig. 1:**
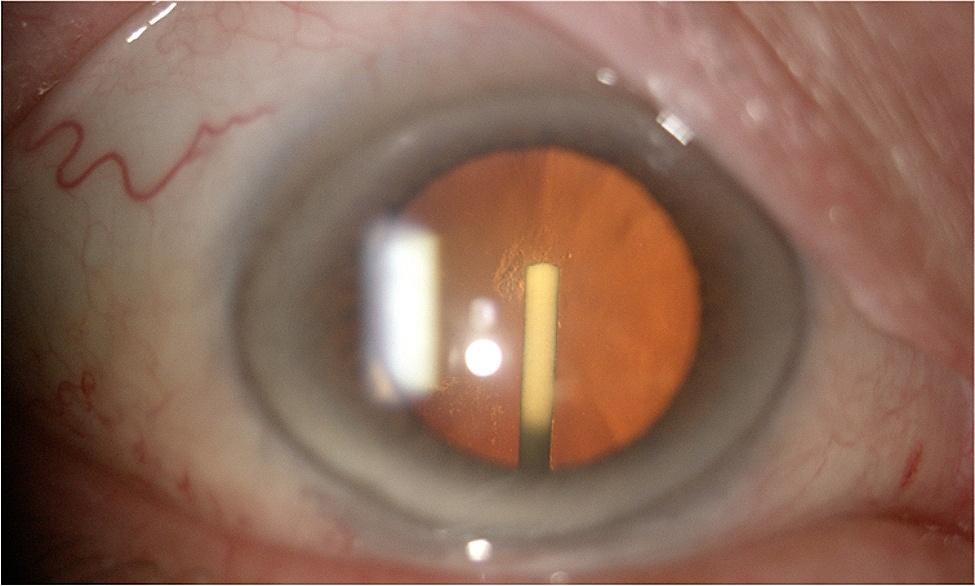
Pre-operative slit lamp photograph showing accidental intralenticular dexamethasone

**Fig. 2:**
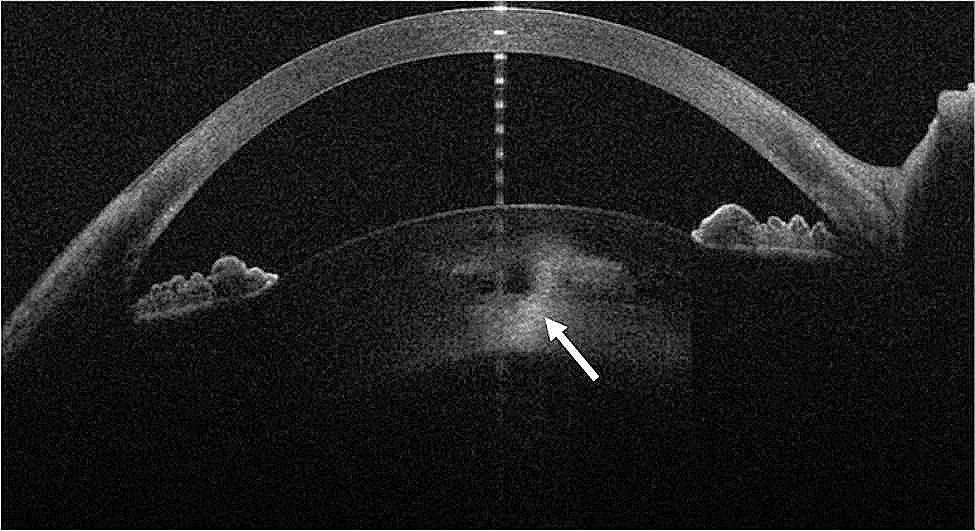
Anterior-segment optical coherence tomography showing the implant location inside the lens (white arrow)

The day after the surgical implantation dexamethasone implant was observed to be located into the lens. (Figures 1 and 2)

Once discussed with the patient, strict follow-up examinations were scheduled at 2, 5 and 15 days post implantation, showing a stable clinical picture. There were neither complications related to the incorrect positioning of the device nor any beneficial effect of dexamethasone on the macular edema. One month after the complication, phacoemulsification with intraocular lens (IOL) implantation and Ozurdex removal was performed.

Under topical anesthesia, a 2.2 mm clear corneal incision and one side port incision were made. Intracameral ophthalmic viscosurgical device (OVD) was injected and after capsulorhexis and gentle hydrodelineation and hydrodissection, nuclear phacoemulsification was performed. Ozurdex was noted to be completely located in the lens. Once the implant became closer, cohesive OVD was smoothly injected in order to isolate the Ozurdex implant. The device was successfully and completely removed using manual forceps, even though it fragmented.

After completing phacoemulsification and automated aspiration of cortical remnants, a posterior capsular rupture with vitreous prolapse and Ozurdex implant remnants were noted, thus, fragments removal by maintaining infusion line in the anterior chamber with an anterior vitrectomy were performed.

After OVD injection, a 3-piece IOL was implanted in the ciliary sulcus. Removal of OVD was performed, and acetylcholine was then injected into the anterior chamber. Pupil was round and centered at the end of surgery. Finally, Intracameral injection of 1 mg Cefuroxime and subconjunctival injection of dexamethasone plus gentamycin were performed (Video 1). Ofloxacin 0.3% and dexamethasone 0.2% eye drops were administered four times per day for 3 weeks and Bromfenac eye drops were administered twice a day for 6 weeks.

**Fig. 3:**
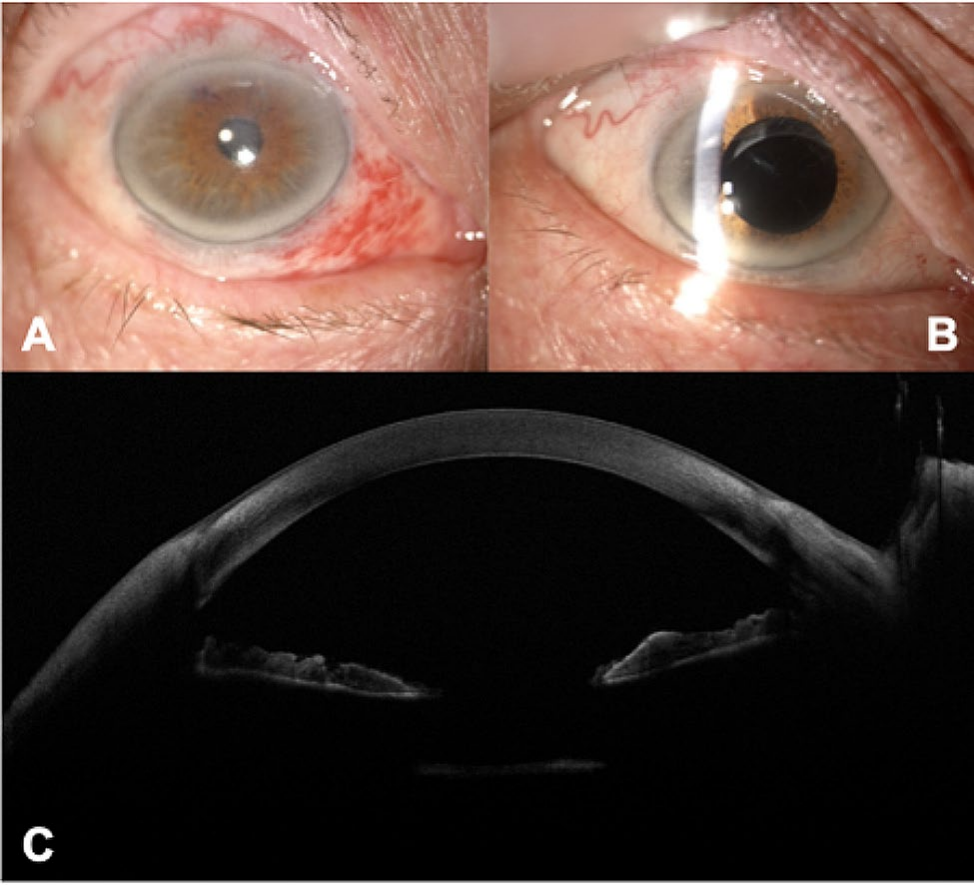
Post-operative Slit lamp photographs before (**A**) and after (**B**) Tropicamide 1% eye drops and Anterior-segment optical coherence tomography (**C**) at one month follow-up

**Fig. 4:**
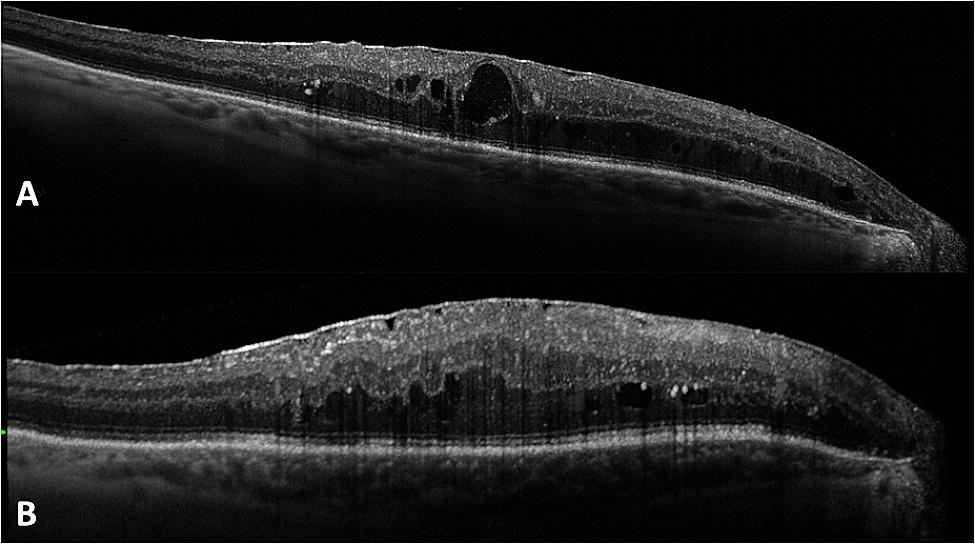
OCT linear macular B scan before (**A**) and after (**B**) surgery, at one month follow up. As evidenced by the OCT scan, the patient was affected by vitreomacular interface disease, which could affect the final visual outcome

Patient was examined 15 days after surgery: BCVA was 0.7 logMAR in his right eye. Intraocular pressure was 16 mmHg. Pupil was round, anterior chamber showed no reaction, IOL was clear and located into the sulcus. Examination at one month confirmed steadiness of the clinical status. (Figures 3 and 4)

## Discussion

We report a case of accidental implant of Ozurdex in the crystalline lens. The condition may derive from an incorrect needle orientation during procedure, surgeon inexperience, or patient movements during procedure, thus it is important to try to prevent this adverse event through a correct head positioning, young colleagues tutoring and with correct needle orientation when performing the procedure through the help of forceps to prevent a wrong globe position. Despite the preventive measures, it is a reported adverse event that therefore needs to be correctly managed. Few cases have been published on this topic, and there is still an open discussion regarding the management of this complication. [1, 2]

Ozurdex may be mainly located into the lens or behind it, and attention must be dedicated to the integrity of the structures embracing the lens (particularly the capsular bag). These considerations may orientate the surgeon towards a conservative or surgical approach.

The first option entails patient observation, consistent with the hypothesis of Berarducci et al., who speculated that even when Ozurdex is not located in the vitreous chamber, the therapeutic effect is maintained through drug release. [1] This evidence has been confirmed by Coca-Robinot et al., who reported slight improvement of the macular condition for 6 months despite the necessity of managing IOP rise complication, which was however reported also in their first case, where Ozurdex removal was performed 1 month after the event. [2]

However, it must be assessed that the conservative approach may expose the patient to rapid cataract development and/or IOP raise. Therefore, close observation is indicated in such cases.

On the other side, an immediate approach consists in a complex surgery, that must not exclude anterior/posterior vitrectomy due to the presence of capsular bag rupture either noted preoperatively or during surgery.

In this case, we decided to remove both the cataract and the Ozurdex one month after the implantation. This because during the observation we did not find any improvement in the retinal disease; therefore, the patient was exposed to the risks of a lens-retained implant without any beneficial effect. This choice is supported by the evidence that the higher concentration of dexamethasone has been shown to be present in both the vitreous humor and the retina between days 7 and 60 after implantation. [3]

A potential drawback of delayed intervention is represented by the risk of implant disruption or fracture during the surgical maneuvers with subsequent release of dexamethasone. This because soon after injection the rigidity of the implant makes it easily removable with forceps. At a later stage, the implant becomes softer. Then the risk that it could split in small fragments is higher. [4]

However, few reports showed that the desegmentation of Ozurdex seems to be innocuous without any immediate or early intraocular complications. [5]

In this report we stress the concept of considering all the variables connected with this uncommon complication being aware that multiple factors are involved in the choice of the proper time for lens and device removal, bearing in mind both patient condition and dexamethasone pharmacokinetic, to guide the choice of time to perform surgery.

### Electronic supplementary material

Below is the link to the electronic supplementary material.

Supplementary Material 1

## Data Availability

All data generated or analyzed during this study are included in this article. Further enquiries can be directed to the corresponding author.

## Abbreviations

BCVA: Best corrected visual acuity
OVD: Ophthalmic viscosurgical device
